# Mechanisms mitigating problems associated with multiple kinetochores on one microtubule in early mitosis

**DOI:** 10.1242/jcs.203000

**Published:** 2017-07-15

**Authors:** Zuojun Yue, Shinya Komoto, Marek Gierlinski, Debora Pasquali, Etsushi Kitamura, Tomoyuki U. Tanaka

**Affiliations:** 1 Centre for Gene Regulation and Expression, School of Life Sciences, University of Dundee, Dundee DD1 5EH, UK; 2 Data Analysis Group, School of Life Sciences, University of Dundee, Dundee DD1 5EH, UK

**Keywords:** Kinetochore, Microtubule, Kinetochore sliding, End-on attachment, Early mitosis, Budding yeast

## Abstract

Proper chromosome segregation in mitosis relies on correct kinetochore interaction with spindle microtubules. In early mitosis, each kinetochore usually interacts with the lateral side of each microtubule and is subsequently tethered at the microtubule end. However, since eukaryotic cells carry multiple chromosomes, multiple kinetochores could occasionally interact with a single microtubule. The consequence of this is unknown. Here, we find that, although two kinetochores (two pairs of sister kinetochores) can interact with the lateral side of one microtubule, only one kinetochore can form a sustained attachment to the microtubule end in budding yeast (*Saccharomyces cerevisiae*). This leads to detachment of the other kinetochore from the microtubule end (or a location in its proximity). Intriguingly, in this context, kinetochore sliding along a microtubule towards a spindle pole delays and diminishes discernible kinetochore detachment. This effect expedites collection of the entire set of kinetochores to a spindle pole. We propose that cells are equipped with the kinetochore-sliding mechanism to mitigate problems associated with multiple kinetochores on one microtubule in early mitosis.

## Abstract (precis)

**Summary:** Given that eukaryotic cells carry multiple chromosomes, multiple kinetochores could occasionally interact with a single microtubule. We identify problems associated with this situation and find mechanisms mitigating these problems.

## INTRODUCTION

**Fig. 1.**
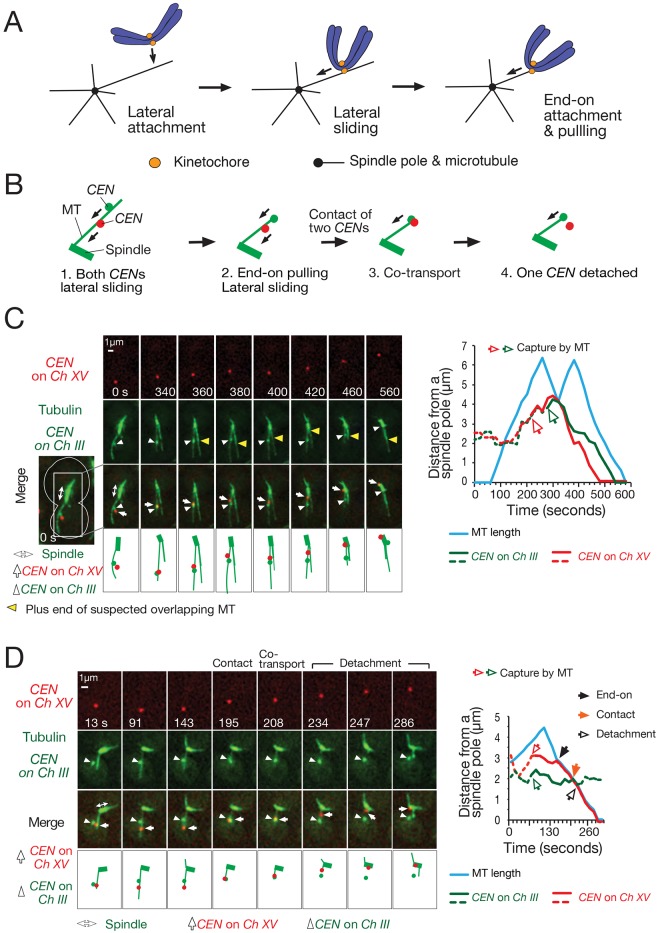
**A single MT accommodates two KTs with lateral attachment but only one KT with sustained end-on attachment.** (A) Diagrams explaining how a KT is captured and transported by a MT in eukaryotic cells. The KT initially interacts with the lateral surface of a single MT (lateral attachment), which extends from a spindle pole; the KT is then transported along the MT lateral surface towards a spindle pole by sliding (lateral sliding). Subsequently, the KT is tethered at the end of a single MT (end-on attachment) and transported polewards as the MT shrinks (end-on pulling) (Tanaka, 2010). (B) Diagrams summarizing the interaction between a single MT and two KTs [two pairs of sister KTs on the indicated centromeres (*CEN*s)]. Two indicated *CEN*s were under control of the *GAL* promoter and visualized as fluorescent dots; these were inactivated, and subsequently reactivated, as in Fig. S1A, to study their interaction with a MT in detail. After both *CEN*s were loaded on the lateral surface of a single MT, they showed sliding along the MT. In some cases, one *CEN* underwent conversion into end-on attachment, was transported by end-on pulling, and subsequently came into contact with the other *CEN*. Then, after brief co-transport, the *CEN* originally proximal to the spindle pole showed detachment. Note that either or both *CEN*s could reach a spindle pole from any of these stages without going through subsequent stages. (C) Representative example in which two KTs showed lateral sliding along a single MT. Cells (T6519) carry *P_GAL_-CEN3-tetOs* (replacing *CEN15* on chromosome *XV*) *TetR-3×CFP P_GAL_-CEN3-lacO* (replacing *CEN3* on chromosome *III*) *GFP-LacI YFP-TUB1 P_MET3_-CDC20*, where *tetO*s are tetracycline operators, TetR is the tetracycline repressor, *lacO*s are lactose operators, and *LacI* is the lactose repressor. The GFP and YFP signals were collected together (green) while CFP signals were acquired separately (red). These cells were treated as in Fig. S1A, i.e. were cultured overnight in methionine drop-out medium with raffinose, treated with a mating hormone for 2.5 h (to arrest cells in G1 phase), and released into fresh media with raffinose, galactose and 2 mM methionine (for Cdc20 depletion and *P_GAL_-CEN* inactivation). After 4 h, cells were suspended in synthetic complete medium containing glucose and methionine (to reactivate *P_GAL_-CEN*). After 10 min incubation, images were acquired every 20 s for 30 min. Time zero is set arbitrarily. *Ch III*, chromosome III; *Ch XV*, chromosome XV. The left panel shows a representative cell while the right shows the profile of KT movement, i.e. graphs of length of the MT that interacted with the two labeled *CEN*s, and positions of those two *CEN*s (distance from a spindle pole; dashed red and green lines represent *CEN*s not on the MT, while solid red and green lines represent *CEN*s on the MT). See Movie 1. T9717 cells (see D) showed similar results (Fig. S1B). (D) Representative example where a laterally attached KT showed detachment after coming into contact with an end-on attached KT. Cells (T9717) with the same genotype as T6519 (see C), except for carrying *GFP-TUB1* instead of *YFP-TUB1*, were treated as in C, and images (GFP and CFP signals) were acquired every 13 s. The graph on right shows the MT length and *CEN* positions as in C. See Movie 2. Another example of KT detachment is shown in Fig. S1B.

For proper chromosome segregation during mitosis, eukaryotic cells need to establish correct kinetochore–microtubule (KT–MT) interactions. This interaction is initiated and developed in a stepwise manner (Cheerambathur and Desai, 2014; Tanaka, 2010). During the early stages of mitosis (prometaphase), a KT (a pair of sister KTs) makes initial contact with the MT lateral surface (lateral attachment; Fig. 1A, left) (Rieder and Alexander, 1990; Tanaka et al., 2005). Once loaded on the MT lateral surface, the KT moves towards a spindle pole by sliding along the MT (Fig. 1A, middle). This KT sliding is promoted by minus-end-directed kinesin (kinesin-14; Kar3 in budding yeast) in budding yeast (*Saccharomyces cerevisiae*) (Tanaka et al., 2007) and probably by KT-associated dynein (and kinesin-14) in vertebrates (Vorozhko et al., 2008; Yang et al., 2007). While the KT undergoes lateral sliding, the KT-associated MT depolymerizes at its distal plus-end; in budding yeast, the speed of this depolymerization is higher than the speed of KT lateral sliding, resulting in the MT plus-end often catching up with a KT attached to its lateral surface (Kitamura et al., 2007; Tanaka et al., 2007). In this event, the KT becomes tethered at the MT plus end (end-on attachment), and moves further towards a spindle pole as MT depolymerization continues at its plus end (end-on pulling; Fig. 1A, right) (Maure et al., 2011; Shrestha and Draviam, 2013). Once KTs are collected on the mitotic spindle, sister KTs can efficiently bi-orient, i.e. interact with MTs extending from opposite spindle poles (Tanaka et al., 2002). All sister KTs must bi-orient prior to chromosome segregation at anaphase.

Poleward KT movement, either by sliding or end-on pulling, is especially crucial when KTs are located at some distance from the mitotic spindle. However, it is unknown why the majority of cells (including budding yeast and vertebrate cells) undergo both sliding and end-on pulling for poleward KT movement. In principle, to transport KTs to a spindle pole, end-on pulling should be sufficient and KT sliding should not be required; i.e. the KT could establish end-on attachment first and then could be transported towards the spindle by end-on pulling as the MT shrinks. In fact, some types of cells, such as fission yeast, undergo KT end-on pulling, but not KT sliding (Franco et al., 2007; Grishchuk and McIntosh, 2006). Is there, then, any advantage of KT sliding in the cells where this mechanism is present?

In both yeast and vertebrate cells, usually each KT (a pair of sister KTs) attaches to the lateral side of a single MT and becomes tethered at the MT end, as mentioned above; subsequently vertebrate KTs interact with multiple MTs (King and Nicklas, 2000). However, since both yeast and vertebrate cells contain multiple chromosomes, two or more pairs of sister KTs could interact with the lateral surface of a single MT during prometaphase, and it is unknown how multiple KTs behave in this situation. For example, can they be transported by lateral sliding on a single MT, and can two (or more) of them establish end-on attachment to one MT? If only one KT is able to establish end-on attachment to one MT, what happens to other KTs on the same MT? Does it cause any problems and, if so, are there any mechanisms to mitigate such problems? In this study, we address these questions using budding yeast as a model organism.

## RESULTS

### A single MT can accommodate only a single KT for sustained end-on attachment, leading to detachment of another KT on the MT lateral surface

To analyze individual KT–MT interactions in detail, we previously developed an engineered assay system in which KT assembly was delayed on a chosen centromere by transcription from an adjacently inserted promoter (Tanaka et al., 2005). This increased the distance between the centromere and the mitotic spindle, and allowed detailed observation of KT–MT interactions after KT assembly on the centromere was induced by turning off the transcription (centromere re-activation assay; Fig. S1A). To address whether two KTs can interact with a single MT, we modified this assay to regulate KT assembly on two centromeres (two pairs of sister centromeres) on different chromosomes (chromosome III and XV). After KT assembly was induced on both centromeres, they were able to interact with the lateral surface of the same or different MTs extending from a spindle pole. We focused on the former case, where both centromeres are caught on the same single MT (Fig. 1B, step 1). A single MT was discerned as reported previously, i.e. by comparing its fluorescent signal with a cytoplasmic MT that is known to be single (Fig. S4 in Tanaka et al., 2005). In these cases, the two centromeres moved by sliding along the MT lateral surface towards a spindle pole (Fig. 1B, step 1; Fig. 1C). Thus, a single MT can accommodate lateral attachment and allow sliding of two KTs.

In some cases, the centromere more distal to the spindle pole was subsequently tethered by end-on attachment to the plus end of a shrinking MT, and continued moving towards the spindle pole by end-on pulling as the MT shrunk (Fig. 1B, step 2; Fig. 1D, 143 s). Such end-on attached centromeres often caught up (came into contact) with the laterally attached more proximal centromere on the same MT (Fig. 1B, step 3; Fig. 1D, 195 s, ‘contact’). In this situation, both centromeres were co-transported poleward at the end of a shrinking MT, for a short distance, following such contact (Fig. 1D, 208 s). Subsequently, the proximal centromere, that had originally been laterally attached prior to the contact, became detached from the MT end (or its proximity), while the original distal centromere continued moving towards the pole by end-on pulling of the shrinking MT (Fig. 1D, 234 and 247 s). Another example of centromere detachment is shown in Fig. S1B. We observed 94 events, in which an end-on attached centromere came into contact with a laterally attached centromere. 28 events out of such 94 events led to centromere detachment. Crucially, in all centromere detachment events, the laterally attached, rather than the end-on attached, centromere showed detachment following the contact. There was no particular bias in the detachment frequency between the two centromeres; the centromeres on chromosome III and XV showed 15 and 13 detachments, respectively. In addition, the speed of co-transport of two KTs (on the two marked centromeres) on average was faster than KT lateral sliding, but slower than KT end-on pulling (Fig. S1C).

It is noteworthy that KT detachment occurred at an approximately constant rate during co-transport after two KTs came into contact (Fig. S1D). The majority of KT detachment (∼80%) happened before KTs had moved more than 2 μm by co-transport. We assume that, after one KT establishes attachment to the end of a depolymerizing MT, another KT may still remain attached at the proximity of the MT end for a short period, and thus be co-transported, but would eventually detach from the MT end (Fig. S1E, left). Alternatively, two relevant chromosomes may be entangled around two KTs for a short period, causing KT co-transport. In any case, if co-transported KTs reached a spindle pole, we were rarely able to detect KT detachment from a pole. We speculate that most KTs that are detached in the immediate vicinity of a spindle pole might be recaptured rapidly by MTs that are in a particularly high density near the pole (Kitamura et al., 2010; Winey et al., 1995), but this would be indiscernible in our assay. There are several short MTs (about 200 nm) extending from the spindle pole (Kitamura et al., 2010; Winey et al., 1995), which would also contribute to rapid recapture of KTs detached in the vicinity of a pole. Alternatively, chromosome crowding in the vicinity of a spindle pole may prevent KTs from dispersing, following their detachment from MTs. In conclusion, we find that a single MT can accommodate only one KT (one pair of sister KTs) for sustained end-on attachment, leading to detachment of other, laterally attached, KTs from the MT plus-ends.

### When sister chromatid cohesion is lost, sister KTs exclude each other from sustained end-on MT attachment

**Fig. 2.**
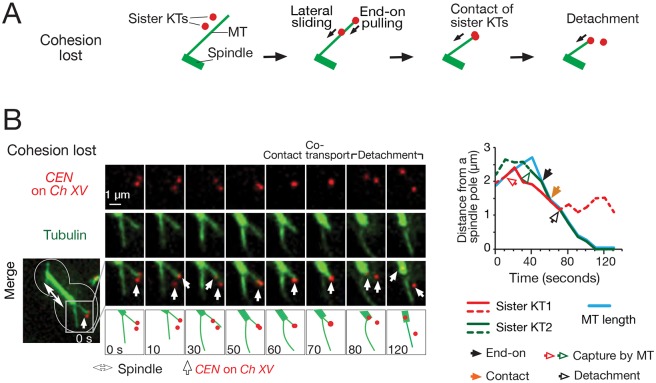
**When sister chromatid cohesion is lost, sister KTs exclude each other from sustained end-on MT attachment.** (A) Diagrams summarizing the interaction of sister KTs with a single MT when cohesion is lost. When cohesion is lost and two sister KTs separate, a laterally attached sister KT detaches from the MT end after coming into contact with an end-on attached sister KT. (B) Representative example where sister KTs interact with a single MT after their cohesion is lost. This interaction was followed by detachment of one sister KT. Cells (T11941) with *Scc1-AnchorAway P_GAL_-CEN3-tetO TetR-3×CFP GFP-TUB1 P_MET3_-CDC20* were treated as in Fig. 1D, except that rapamycin was added upon release from G1 arrest (to deplete Scc1). Images (GFP and CFP signals) were acquired every 10 s. The graph (right) shows the MT length and the position of *CEN*s, as in Fig. 1C. Note that the spindle elongates after Scc1 depletion, although cells are arrested in metaphase (Tanaka et al., 2000). See Movie 3.

The above results suggest that there is a limited capacity of the KT to form an end-on attachment. One KT (one pair of sister KTs) seems to form an ‘exclusive’ attachment to the MT end. We set out to determine what comprises such an exclusive attachment, and whether both sister KTs are involved or whether one sister KT is sufficient to achieve it. Sister KTs are normally connected by sister chromatid cohesion at the centromere region (Tanaka et al., 2013). If this cohesion is lost, sister KTs separate from each other, but each sister can still interact with a MT (Tanaka et al., 2000). To address whether sister KTs prevent each other from forming end-on attachments on a single MT, as do two pairs of sister KTs, we depleted the cohesin subunit Scc1 (also called Mcd1) and investigated how such separated sister KTs interact with MTs. We used the centromere reactivation assay to analyse individual KT–MT interactions in detail in this condition (Fig. S1A). We focused on situations where two sister KTs were initially caught on the lateral surface of the same MT (Fig. 2A). Subsequently one sister KT, usually the one distal to the spindle pole, was often ‘tethered’ at the MT end and moved towards a spindle pole as the MT shrunk, indicating end-on attachment (Fig. 2B, 50 s). This end-on attached KT then caught up with its sister on the MT lateral surface (60 s, ‘contact’), which led to detachment of one sister KT from the MT end (80 s). In total, we observed 17 examples of sister KT detachment (following 52 contact events). As the two sister KTs could not be distinguished in this situation, we were not certain which sister KT showed detachment. Nonetheless, we assumed that it was the KT originally on the MT lateral surface that showed detachment, based on our analogous observation of two pairs of sister KTs (Fig. 1B,D). In conclusion, if cohesion is lost, two separate sister KTs prevent each other from forming the end-on MT attachment.

The results so far suggest that there is a limited capacity of the KT to form sustained end-on attachment. In fact, once one KT (pair of sister KTs) forms an end-on attachment, another KT cannot form a sustained end-on attachment on the same MT, and any KT on the MT lateral surface shows detachment after it comes into contact with an end-on attached KT (Fig. 1B,D). The same thing happens if two sister KTs separate from each other due to a loss of cohesion, i.e. once one sister KT forms an end-on attachment, it prevents the other sister from making a sustained end-on attachment (Fig. 2A,B). Thus, a single sister KT is sufficient to establish ‘exclusive’ end-on attachment (Fig. S1E, right). One possible interpretation of the limited KT capacity for the attachment to the MT end is that, once a single KT attaches at the MT end, it takes up MT-binding sites with a strong affinity and thus sterically excludes other KTs from achieving a high-affinity stable attachment.

### KT lateral sliding along a MT delays and diminishes discernible KT detachment caused by a contact with another KT at the MT end

As shown above, a KT on the MT lateral side shows detachment if it comes into contact with another KT that is attached to the end of the same MT. If such a detachment happens frequently, it could compromise efficient KT collection to the spindle, or to a spindle pole in yeast. Is there any mechanism to mitigate such adverse effects? We envisaged that KT lateral sliding along the MT might contribute to such mitigation by moving laterally attached KTs towards a spindle pole before end-on attached KTs come into contact with, and detach, them. In budding yeast, lateral sliding of a KT is driven by the minus-end-directed kinesin, Kar3 (a kinesin-14 member), which localizes at the KT (Tanaka et al., 2007, 2005). To address the effects of a lack of KT sliding, we depleted Kar3 and compared the number of KT detachments with those in Kar3 wild-type cells. We used the centromere reactivation assay (Fig. S1A) with two reactivated centromeres. To obtain the number of samples required for optimal statistical analysis, we conducted the experiments in a Slk19-depletion background, which diminished the association between the two marked centromeres (two pairs of sister centromeres) in this experimental setting (Richmond et al., 2013). Slk19 depletion did not affect the KT sliding function of Kar3 (Fig. S2A).

**Fig. 3.**
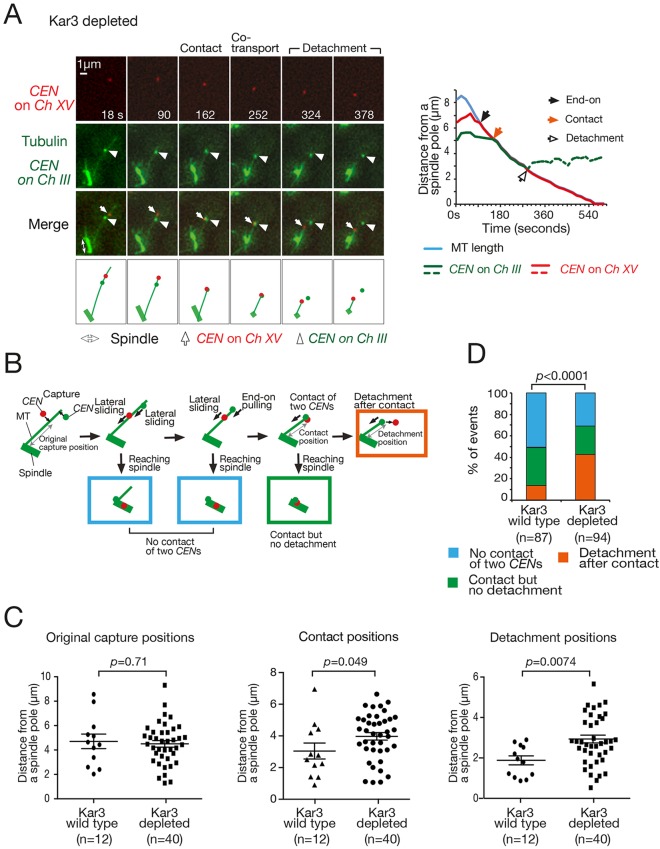
**KT lateral sliding along a MT diminishes discernible KT detachment that occurs after coming into contact with an end-on attached KT.** (A) Representative example of a Kar3-depleted cell where a laterally attached KT showed detachment after coming into contact with an end-on attached KT. Cells (T11469) carrying *kar3-aid slk19-mini-aid TIR P_GAL_-CEN3-tetOs* (replacing *CEN15* on chromosome *XV*) *TetR-3×CFP P_GAL_-CEN3-lacO* (replacing *CEN3* on chromosome *III*) *GFP-LacI GFP-TUB1 P_MET3_-CDC20* were treated as in Fig. 1C, except that 1-naphthaleneacetic acid (NAA) was added to deplete Kar3 and Slk19 when cells were released from G1 arrest. Images (GFP and CFP signals) were acquired every 18 s. The graph (right) shows the MT length and the position of *CEN*s, as in Fig. 1C. See Movie 4. (B) Diagrams explaining analyses in C and D. ‘Original capture position’, ‘contact position’ and ‘detachment position’ were measured as shown here and plotted in C. Rectangles in color represent the categorized situations shown in D in the same color. (C) In the absence of KT sliding, contact between two *CEN*s and subsequent detachment of the *CEN* happen further from a spindle pole. Graphs show the initial capture positions (distance from a spindle pole) for *CEN*s that subsequently showed detachment (left, see B), positions of end-on attached *CEN* coming into contact with a laterally attached *CEN* (middle; see B) and the positions of *CEN* detachments (right; see B). T11469 cells (see A) and T11497 cells (*KAR3+ slk19-mini-aid*, otherwise the same as T11469 cells) were treated and images were acquired as in A. Graphs show individual data points and mean±s.e.m. *P*-values were obtained by *t*-test. (D) In the absence of KT sliding, *CEN* detachment is observed more frequently. Following the situation where two *CEN*s formed a lateral attachment on the same MT, one of the following three events took place (see B): (1) both *CEN*s reached a spindle pole without one coming into contact with the other (blue), (2) after one C*EN* formed end-on attachment, it came into contact with the other *CEN* and the two *CEN*s were co-transported to the spindle pole (green), or (3) after one C*EN* formed end-on attachment, it came into contact with the laterally attached *CEN*, which subsequently detached from the MT end (orange). Images acquired in A were used for this analysis. The graph shows the percentage of each event. The *P*-value was obtained by use of a *χ*-squared test for trend (the order for the trend was blue, green and orange).

As expected, KT sliding was abolished after Kar3 depletion (Fig. S2A). In both Kar3-depleted and Kar3 wild-type cells, a laterally attached KT showed detachment after coming into contact with an end-on attached KT; Fig. 3A shows an example of a Kar3-depleted cell. The rate of KT detachment after the contact was similar in the two cells (Fig. S2B). We then analyzed the position (distance from a spindle pole) of (1) the initial KT capture by a MT, (2) an end-on attached KT coming into contact with a laterally attached KT, and (3) subsequent detachment of a laterally attached KT from the MT end (Fig. 3B). A KT was caught on the lateral side of a MT at similar distances from a spindle pole in both Kar3 wild-type and Kar3-depleted cells (Fig. 3C, left). However, in Kar3-depleted cells, end-on attached KTs came into contact with laterally attached KTs further away from a pole (Fig. 3C, middle). In these cells, the KT detachment was detected more frequently, and at a greater distance from the spindle pole, than in Kar3 wild-type cells (Fig. 3C, right, D). As we discussed in the first section, we reason that many KTs, detached in the immediate vicinity of a spindle pole might be indiscernible since they are often recaptured rapidly by MTs whose density is high in that region (Kitamura et al., 2010; Winey et al., 1995). It is therefore likely that, in Kar3-depleted cells, more KT detachments following contacts occur at a greater distance from a spindle pole, which would make discernible detachments more frequent. We conclude that KT lateral sliding along a MT towards a spindle pole delays and diminishes discernible KT detachment after an end-on attached KT comes into contact with a laterally attached KT.

### KT lateral sliding along a MT also delays and diminishes discernible KT detachment caused by a contact with another KT at the MT end in physiological conditions

So far we have used the centromere reactivation assay to study how two KTs interact with the same single MT (Fig. S1A). We next addressed the same question in physiological conditions, without using the centromere reactivation assay and without using Slk19 depletion. In physiological conditions, KTs are attached to MTs during most of the cell cycle in budding yeast (Winey and O'Toole, 2001). However, upon centromere DNA replication KTs at least partially disassemble, leading to detachment of centromeres from MTs (Kitamura et al., 2007). Kinetochores are reassembled and interact with MTs again within 1–2 min, making initially lateral, and then end-on attachment (Kitamura et al., 2007).

**Fig. 4.**
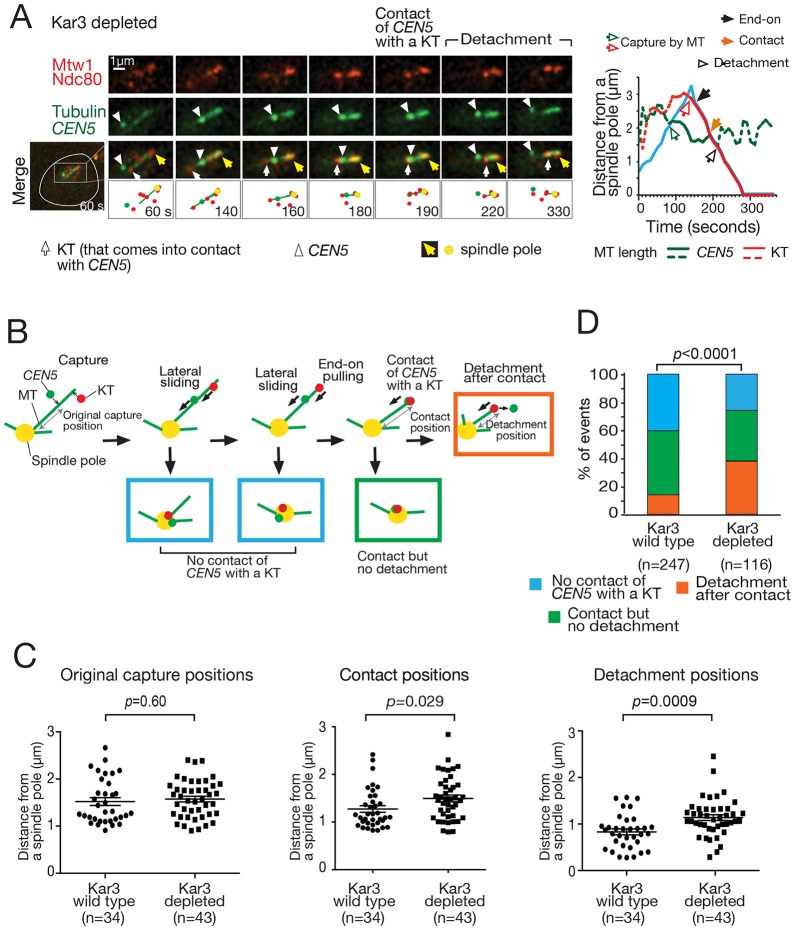
**KT detachment is found in physiological conditions after an end-on attached KT comes into contact with a laterally attached KT.** (A) Representative example of centromere detachment in a Kar3-depleted cell in physiological conditions. Cells (T11434) with *kar3-aid TIR1 CEN5-lacOs GFP-LacI GFP-TUB1 MTW-4×mCherry NDC80-4×mCherry* were treated with a mating pheromone for 3 h (to arrest them in G1 phase) and released into fresh medium. NAA was added for the last 30 min of the G1 arrest and also after release from the arrest (to deplete Kar3). 30 min after release from the G1 arrest, images (GFP and mCherry signals) were acquired every 10 s. See Movie 5. A representative example of a Kar3 wild-type cell is shown in Fig. S3B. (B) Diagrams explaining analyses in C and D. The ‘original capture position’, ‘contact position’ and ‘detachment position’ were measured as shown here and plotted in C. Rectangles in color represent the categorized situations shown in D in the same color. (C) In the absence of KT sliding, contact between a KT (not at *CEN5*) and *CEN5*, and subsequent detachment of *CEN5* happens further from a spindle pole. Graphs show the initial capture position (distance from a spindle pole) of *CEN5* only in cases where there was subsequent detachment (left; see B), the position of KTs coming into contact with *CEN5* (middle; see B) and the positions of *CEN5* detachment (right; see B). T11434 cells (see A) and T11435 cells (*KAR3+*, otherwise the same as T11434 cells) were treated and images were acquired, as in A. Graphs show individual data points and mean±s.e.m. The *P*-values were obtained by *t*-test. (D) In the absence of KT sliding, *CEN5* detachment happens more frequently in physiological conditions. Images acquired in A were used for this analysis. Following the situation where a KT (not on *CEN5*) and *CEN5* formed a lateral attachment on presumably the same MT (the KT is more distal to a spindle pole than *CEN5*), one of the following three events took place (see B): (1) both the KT and *CEN*5 reached a spindle pole without contact (blue), (2) after the KT formed end-on attachment, it came into contact with *CEN5* and they were co-transported to the spindle pole (green), or (3) after the KT formed end-on attachment, it came into contact with *CEN5*, and *CEN5* showed detachment (orange). Images acquired in A were used for this analysis. The graph shows the percentage of each event. The *P*-value was obtained by use of a *χ*-squared test for trend as in Fig. 3D.

We visualized one centromere and KTs, and analyzed the cases where the marked centromere and one KT (on another centromere) interacted with presumably the same MT (see Materials and Methods). We focused on the cases where the centromere was proximal, and the KT distal, to a spindle pole on the same MT (see Fig. 4A, 140–180 s). We chose such cases for our analyses because of the reasons explained in Fig. S3A. Figs S3B and Fig. 4A show examples of a Kar3 wild-type and Kar3-depleted cell, respectively. We confirmed that in Kar3-depleted cells the centromere did not show sliding to a spindle pole, as expected (Fig. S3C). After the KT on the MT end came into contact with the centromere (Fig. 4A, 190 s), the centromere detached from the MT end (220 s), which is similar to what we observed in the centromere reactivation assay. The rate of centromere detachment after the contact events was similar in Kar3 wild-type and Kar3-depleted cells (Fig. S3D). We then compared the position (distance from a spindle pole) of the centromere upon the following key events (Fig. 4B). In Kar3 wild-type and Kar3-depleted cells, the centromere was caught at similar distances from a spindle pole (Fig. 4C, left). However, in Kar3-depleted cells, laterally attached centromere came into contact with end-on attached KTs further away from a pole than in Kar3 wild-type cells (Fig. 4C, middle). Then, in Kar3-depleted cells, the centromere detachment following the contact happened more frequently and further from a pole (Fig. 4C right, D). As discussed in the previous section, we speculate that detachment of centromeres in the vicinity of a spindle pole might often be indiscernible because they are quickly recaptured by MTs whose density is high around a spindle pole. We conclude that, in physiological conditions, KT lateral sliding along a MT delays and diminishes discernible KT detachment caused by a contact with an end-on attached KT.

### Lateral KT sliding shortens the time required for collecting the complete set of KTs to a spindle pole by delaying KT detachments

**Fig. 5.**
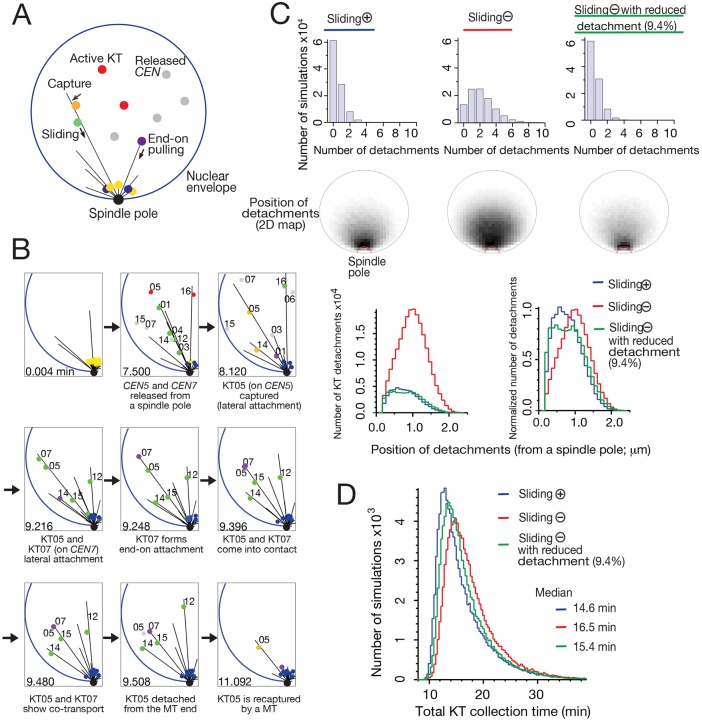
**Mathematical simulation shows that KT lateral sliding along MTs shortens the total KT collection time by delaying and diminishing discernible KT detachments.** (A) Diagram outlining a computer simulation that recapitulates the initial KT–MT interaction, projected on the *x-z* plane (Gandhi et al., 2011). KTs locate in the vicinity of a spindle pole before centromere (*CEN*) DNA replication (yellow dots). Upon *CEN* replication, KTs disassemble, and *CEN*s move away from a pole (gray dots) (Kitamura et al., 2007). KTs are then reassembled (red dots) on *CEN*s, interact with the lateral side of MTs extended from a spindle pole (orange dots) and slide along a MT towards a spindle pole (green dots). KTs are then tethered at the MT end and transported polewards by MT end-on pulling (purple dots). Subsequently, they are tethered at the end of short MTs in the vicinity of the pole (blue dots). Representative examples of computer simulations are shown in Movies 6 and 7. (B) Example of KT detachment after an end-on attached KT comes into contact with a laterally attached KT; in the absence of KT sliding, projected onto the *x-z* plane. At 9.396 min, an end-on attached KT (KT07, KT on *CEN7*) came into contact with a laterally attached KT (KT05, KT on *CEN5*). After co-transport for a short period, the laterally attached KT05 showed detachment at 9.508 min. (C) Frequency and positions of KT detachments. 100,000 simulations were carried out in each of the following three conditions: the presence (wild-type condition) and absence of sliding, and no sliding with reduced detachments (9.4% of ‘standard’ rate). In each condition, the graph (top) shows the number of simulations (*y*-axis) with the indicated number of KT detachments (*x*-axis), the two-dimensional density maps (middle) show the positions of KT detachments, projected onto the *x-z* plane, and the graph on the bottom left shows the numbers of KT detachments (*y*-axis) that happened at the given distance from a spindle pole (x-axis), categorized in each bin (0.09 μm); numbers of KT detachments are also shown (bottom, right) after normalization (the maximum number was normalized to 1.0 in each condition). (D) Total KT collection time, i.e. the time from the first centromere detachment from a spindle pole until the last centromere reached a pole and formed the end-on attachment, was analyzed in three conditions shown in C. A total of 100,000 simulations were carried out in each condition. Graph shows the number of simulations (*y*-axis) with the total KT collection time (*x*-axis), categorized in each bin (0.32 min interval).

The detachment of laterally attached KTs, after coming into contact with end-on attached KTs, may delay collection of all KTs to a spindle pole, which could then compromise the fidelity of subsequent bi-orientation establishment (see Discussion). We next aimed to evaluate how the KT detachment affects overall KT collection to a spindle pole, but it was difficult to address this using live-cell imaging because we could not visualize all the KTs; the intensity of some KTs was too weak to observe (Kitamura et al., 2007). We therefore employed a mathematical simulation (see Materials and Methods). We simulated the following process (Fig. 5A): a yeast cell carries 16 chromosomes, and all 16 centromeres are tethered to short MTs (100–200 nm) in the vicinity of a spindle pole in G1 phase (Kitamura et al., 2010; O'Toole et al., 1999). Upon DNA replication, KTs disassemble and centromeres move away from a spindle pole (Kitamura et al., 2007). Within 1–2 min KTs reassemble, allowing centromeres to again interact with MTs, making lateral attachment initially and then end-on attachment. Subsequently, KTs slide along MTs and move further by end-on pulling towards a spindle pole. If an end-on KT comes into contact with a laterally attached KT on the same MT, the lateral KT shows detachment after KT co-transport for a short period (Fig. 5B), as we found in live cells, above. For the simulation, the average speed of KT displacement along a MT was estimated from the results of live-cell imaging in Fig. S4A, and other parameter values for MT dynamics and KT motions were obtained from previous studies (Gandhi et al., 2011; Kalinina et al., 2013; Kitamura et al., 2007, 2010; Tanaka et al., 2007).

Using this simulation, we ‘switched off’ the KT sliding and compared the outcome with that from the ‘wild-type’ condition in which KT sliding was normal. In the absence of sliding, we found that ‘discernible’ KT detachment happened more frequently and at a greater distance from a spindle pole, after coming into contact with an end-on attached KT (Fig. 5C; sliding plus and minus, compare blue and red). These results are consistent with the results of live-cell imaging in physiological conditions (see Fig. 4C,D). Note that, in the simulation, we defined ‘discernible’ KT detachment as a minimum of 30 s before subsequent recapture by a MT, since we could not detect recapture in less than 30 s by live-cell imaging. The simulation also largely recapitulates both the position and frequency of KT detachments in live-cell imaging (Fig. S4B,C). Next, we compared the total KT collection time, i.e. the time from the first centromere detachment from a spindle pole until the last centromere reached a pole and formed an end-on attachment. In the absence of sliding, the distribution of the total KT collection time was shifted towards the right (Fig. 5D; sliding plus and minus, compare blue and red). Thus, in the simulation, KT sliding enhances the efficiency of KT collection to a spindle pole and shortens total KT collection time.

KT sliding along a MT could shorten the total KT collection time either by bringing KTs more rapidly towards a spindle pole or by diminishing KT detachment (as a result of delaying contact between end-on and laterally attached KTs). To address which effect contributes most to shortening the total KT collection time, we analysed total KT collection time with the simulation after making KT detachment frequency without KT sliding similar to that with KT sliding. The KT detachment frequency became similar with and without KT sliding when the parameter value defining the KT detachment rate was reduced to 9.4% without KT sliding (Fig. 5C, compare green and blue). Intriguingly, when KT sliding was absent, the reduced detachment led to a shift of total KT collection time to the left (Fig. 5D, shift from red to green). This suggests that lateral KT sliding reduces total KT collection time by, at least partly, diminishing KT detachment. In comparison, diminishing KT detachment seems to contribute more to shortening total KT collection time (shift from red to green in Fig. 5D) than does bringing KTs more rapidly to a spindle pole (shift from green to blue in Fig. 5D). After frequent KT detachment in the absence of KT sliding, some detached centromeres require a long time for recapture, which leads to a prolonged total KT collection time (Fig. S4D–G). In conclusion, the simulation suggests that the KT lateral sliding along MTs diminishes the KT detachment caused by contact with end-on attached KTs, and thus contributes to shortening total KT collection time.

## DISCUSSION

Proper chromosome segregation in mitosis relies on chromosome bi-orientation, i.e. attachment of sister KTs to the ends of MTs extending from the opposite spindle poles (Kalantzaki et al., 2015). How could KTs establish bi-orientation efficiently? A KT (pair of sister KTs) initially interacts with the MT lateral surface, which provides a much larger contact surface than does the MT end. This ensures an efficient encounter between the KT and a MT (Rieder and Alexander, 1990; Tanaka et al., 2005). The KT then needs (1) to establish attachment to the MT end (end-on attachment) and (2) to be transported to the vicinity of a spindle pole (KT collection) in budding yeast (where the bipolar spindle is subsequently formed) or to the center of the spindle in metazoan cells, where many MTs extend from both spindle poles at high density (Kitamura et al., 2007; Shrestha and Draviam, 2013; Tanaka et al., 2007). To do so, in principle the KT could establish end-on attachment first and then could be transported towards the spindle as the MT shrinks (end-on pulling). Indeed, this strategy could work well in a cell with only a small number of chromosomes. However, our results suggest that if cells have many chromosomes they need the second mechanism of KT transport for efficient KT collection. In fact, the MT end can accommodate only one KT (pair of sister KTs) for sustained end-on pulling, and if multiple KTs are on the same MT they detach from the MT end except for the first KT to form an end-on attachment (Fig. 1B,D). Frequent detachments prolong the time required for collection of the complete set of KTs (Fig. 5D). To reduce the frequency of detachments, a KT can be transported, by sliding, along a MT towards the spindle (or spindle pole) before the end of a shrinking MT (on which another KT could be attached) reaches it, i.e. the lateral sliding can delay and diminish discernible KT detachment. This explains why vertebrate cells and budding yeast are equipped with a mechanism for promoting KT sliding along a MT; i.e. in these cells, the MT minus-end-directed motors dynein and Kar3 (kinesin-14), localize at KTs and drive KT sliding (Tanaka et al., 2007; Vorozhko et al., 2008; Yang et al., 2007).

Human cells and the budding yeast *S. cerevisiae* (diploid in the natural environment) carry 46 and 32 chromosomes, respectively. However, in cells with far fewer chromosomes, KT detachments would be rare even without KT sliding. Therefore, if the major role of KT sliding were indeed to diminish KT detachments, we would postulate that KTs might not undergo sliding along a MT in cells with fewer chromosomes. Fission yeast *Schizosaccharomyces pombe* (haploid in the natural environment) carries only three chromosomes, and this organism notably lacks a mechanism of KT sliding along a MT (Franco et al., 2007; Grishchuk and McIntosh, 2006). In *S. pombe*, the kinesin-14 member Klp2 still localizes at KTs (Gachet et al., 2008; Grishchuk and McIntosh, 2006), but may have lost the ability to drive KT sliding along a MT while the number of chromosomes was reduced during the evolution of *S. pombe* (Dujon, 2010). It will be intriguing to address whether KT sliding along a MT is present or absent in more organisms carrying a variety of numbers of chromosomes.

Meanwhile, in vertebrate cells, it is still unclear how frequently two or more kinetochores could attach to one MT in early mitosis and how frequently an end-on attached KT comes into contact with a laterally attached KT. In any case, vertebrate KTs are larger than budding yeast KTs and may show a greater steric exclusion once end-on attachment has been established. For example, an end-on attached KT may more readily exclude a laterally attached KT when they come into contact in vertebrate cells, leading to a quicker detachment (i.e. after a shorter period of co-transport) of the laterally attached KT than in budding yeast. Nonetheless, dynein can drive KT sliding at a higher speed in vertebrate cells than does Kar3 (Tanaka et al., 2007; Vorozhko et al., 2008; Yang et al., 2007); thus discernible KT detachment might be more effectively diminished in vertebrate cells. How vertebrate cells mitigate problems associated with multiple KTs on one MT in early mitosis is an important research topic.

## MATERIALS AND METHODS

### Yeast strains and cell culture

The background of yeast strains (W303) and the methods for yeast culture have been described previously (Amberg et al., 2005; Tanaka et al., 2007). The genotypes of strains used in this study are shown in Table S1. To synchronize cells in the cell cycle, yeast cells were arrested in G1 phase by treatment with yeast mating pheromone (α- or a-factor) and subsequently released into fresh medium (Amberg et al., 2005; O'Reilly et al., 2012). The a-factor was synthesized as reported previously (O'Reilly et al., 2012). Cells were cultured at 25°C in YPA medium containing 2% glucose (YPAD) unless otherwise stated. To activate the *GAL* promoter, cells were pre-incubated in medium containing 2% raffinose (without glucose) for at least for 3 h, and subsequently incubated in medium containing both 2% galactose and 2% raffinose. Cells were incubated in medium containing 2% glucose to suppress the *GAL* promoter (without subsequent activation). The *MET3* promoter was activated by incubation of cells in methionine drop-out medium, and suppressed by adding 2 mM methionine to the relevant media. Constructs *CEN15-tetOs*, *CEN5-tetOs* (Tanaka et al., 2000), *P_GAL_-CEN3-tetOs* (Hill and Bloom, 1987; Michaelis et al., 1997; Tanaka et al., 2005), *TetR-3*×*CFP* (Bressan et al., 2004; Michaelis et al., 1997), *P_MET3_-CDC20* (Uhlmann et al., 2000), *GFP-TUB1* (Straight et al., 1997), were as described previously. *P_GAL_-CEN3-lacOs* was constructed similarly to *P_GAL_-CEN3-tetOs* (to replace *CEN3* on chromosome III) but designed to replace *CEN15* on chromosome *XV*; this was visualized with GFP–LacI that bound *lacO*s (Straight et al., 1996). The *pDH20* plasmid containing *YFP-TUB1* was obtained from Yeast Resource Center (Seattle). The *NDC80* and *MTW1* genes were tagged with 4*×mCherry* at their C-terminus at their original gene loci by a one-step PCR using a 4*×mCherry* cassette (pT909) as a PCR template (Maure et al., 2011).

### Centromere reactivation assay

To analyze individual KT–MT interactions in detail, the centromere re-activation assay was used (Tanaka et al., 2010, 2005). In this assay, KT assembly was delayed on a chosen centromere (*CEN3-tetOs* or -*lacOs*, replacing *CEN3* on chromosome III and/or *CEN15* on chromosome XV) by inducing transcription from the *GAL* promoter. This increased the distance between the centromere and the mitotic spindle, allowing detailed observation of KT–MT interactions after inducing KT assembly on the centromere by turning off the *GAL* promoter in metaphase arrested cells (Fig. S1A). Cells with *P_GAL_-CEN3-tetOs* (or *-lacOs*) *P_MET3_-CDC20* (see full genotypes in Table S1) were cultured as explained in the legend of Fig. 1C.

### Analysis of KT–MT interaction in physiological conditions

In our study of the initial KT–MT interaction in physiological conditions (Fig. 4), we visualized one centromere and KTs because of the technical reasons explained in Fig. S3A. We analyzed the cases where the centromere and one KT (on another centromere) were on the same line of a MT signal (whose intensity is uniform along the line) extending from a spindle pole. In these cases, we reasoned that the visualized centromere and the KT of our interest are on the same MT, at least in the majority of the cases (even if not all cases). Supporting this notion, end-on pulling showed a higher velocity than did lateral sliding (Fig. S3C), as found in the centromere reactivation assay (Fig. S1C), where a single MT is more easily discernible; we would not expect this result if we often failed to discern single MTs and thus often mixed up end-on pulling with the lateral sliding.

### Depletion of Scc1, Kar3 and Slk19

To deplete Scc1 protein, an anchor away system was used (Haruki et al., 2008); this consists of *SCC1–FRB* (C-terminal tag at the original *SCC1* locus), *RPL13A-2×FKBP12, TOR1-1* and *fpr1*Δ. In the presence of rapamycin (10 µM), Scc1 protein bound Rpl13A ribosomal protein due to the FRB–FKBP12 interaction, which leads to depletion of Scc1 in the nucleus. To deplete Kar3 and Slk19, *KAR3* and *SLK19* were tagged with *aid* and *mini-aid* tags (auxin-inducible degron tags), respectively, at their C-termini at original loci in a strain carrying the rice F-box gene *TIR1* (Kubota et al., 2013; Nishimura et al., 2009). In the presence of the auxin naphthaleneacetic acid (NAA; 1 mM), *aid*-tagged proteins bind Tir1, leading to their ubiquitylation and degradation.

### Live-cell imaging and image analyses

The procedures for time-lapse fluorescence microscopy were as described previously (Tanaka et al., 2010). Time-lapse images were collected at 25°C. Images were acquired using a DeltaVision Core or Elite microscope (Applied Precision), an UPlanSApo 100× objective lens (Olympus; NA 1.40), SoftWoRx software (Applied Precision) and a CoolSnap HQ camera (Photometrics). We acquired 7–9 (0.7 µm apart) *z*-sections, which were subsequently processed through deconvolution, and analysed with Volocity (Improvision) software. CFP, GFP, and mCherry signals were discriminated using the 89006 multi-band filter set (Chroma). For the image panels in figures, *z*-sections were projected to two-dimensional images. Statistical analyses were carried out using Prism (Graph Pad) software.

### Computer simulation of KT–MT interaction

We created a computer model and carried out simulations of the initial KT–MT interaction, based on the configuration in physiological conditions (Fig. 4; Kitamura et al., 2007). The simulation was previously developed (Gandhi et al., 2011; Vasileva et al., 2017), but several modifications were introduced in this study. The values of the majority of parameters were determined, based on previous studies (Gandhi et al., 2011; Kalinina et al., 2013; Kitamura et al., 2007, 2010; Tanaka et al., 2007, 2005), and some unknown parameter values were determined in the current study (Table S2).

The model was computed as a series of events on a constant time step Δ*t*. All objects (MTs, KTs and Stu2) were located in a three-dimensional space. The nucleus was represented by a sphere of radius *R*_nuc_. An exclusion radius, *r*_ex_, was established around the spindle pole. Each MT was a line segment extending into the nucleus from the spindle pole. Each KT was a point inside the nucleus. MTs could grow and shrink with speed *v*_gro_ and *v*_shr_, respectively. Parameters defining MT dynamics, such as the initial MT number (*n_MT_*), MT catastrophe rate (*K_cat_*) and MT beaming factor β, were set as in Gandhi et al. (2011). When a growing MT hit the nuclear envelope, it started to shrink. When an empty MT shrank to *r*_ex_, it could start growing at a certain nucleation rate *K*_nuc_, unless there were KTs waiting at *r*_ex_, in which case the MT captured the KT and showed no further change. The MTs also experienced ‘pivoting’, which was modeled by angular random walk with the diffusion coefficient *D*_MT_ (Brun, 2011; Kalinina et al., 2013). Stu2 was a MT polymerase that causes MT rescue (Gandhi et al., 2011) and its properties [Stu2 sending rate (*K_stu2_*), Stu2 speed (*v_stu2_*), probability of MT rescue (*P_re_*_s_) and KT rescue delay (*t_d_*)] were defined as in Gandhi et al. (2011). Time 0 was defined as the mean time of replication of the first centromere (*CEN2*) (Vasileva et al., 2017). When replicated, a centromere detached from a pole and could move freely by a random walk with diffusion coefficient *D*. After a delay (*t*_del_), a KT was reassembled at the position of the centromere.

KTs also moved inside the nucleus (but not within the exclusion radius) with a diffusion coefficient *D*. Once attached to a MT, a KT moved laterally along a MT towards the spindle pole or was pulled by the distal end of the MT with speed *v*_lat_ or *v*_pul_, respectively. Sliding motion was varied by a linear diffusion with a coefficient *D*_lat_. When a sliding KT reached the exclusion radius *r*_ex_, it remained there until an empty MT shrank to *r*_ex_. Then, the KT was caught at the end of this MT; no further change occurred to such a KT and MT, apart from MT pivoting. The same happened immediately when an end-on pulled KT reached *r*_ex_.

The interaction between KT-generated and spindle MTs was simplified by assuming a certain capture radius, *R*_KT_, around each KT. If a KT was found at a distance *R*_KT_ from any part of a spindle MT, the KT-derived MT connected to this spindle MT over the shortest distance and brought the KT towards the spindle MT, usually on its lateral side, at a speed *v*_cap_. Once capture was completed, the KT began sliding, which was converted to end-on pulling if end-on attachment was subsequently established. The simulation was completed once all 16 reassembled KTs reached *r*_ex_ and established end-on attachment.

If an end-on pulled KT came into contact with another KT that was sliding along the same MT, they went into ‘co-transport’ mode. Both KTs traveled together at speed of *v*_tran_ while the sliding KT could detach (detachment) at a rate *K*_evi_. In rare events where an end-on pulled KT came into contact with two (or more) other KTs on the lateral side of the same MT, *K*_evi_ was applied separately for the two others. We assumed that the detached KT was not able to re-attach to a MT until MTs grew from the KT and reached the length *R*_KT_; i.e. for *R*_KT_/*v*_gro_ (in minutes) (KT-derived MTs showed a similar growth rate to spindle MTs; Kitamura et al., 2010).

The code for the simulation was written in Perl and simulations were run in a Linux environment. We ran 100,000 individual simulations in each condition. Detachments were counted and analyzed only if it took more than 0.5 min for detached KTs to be recaptured by a MT extending from a spindle pole; this is because KT detachment times of less than 0.5 min were difficult to recognize in live-cell imaging. To switch off KT sliding, the average KT displacement speed (which is normally 0.6 μm/min) was set at 0. To reduce the KT detachment frequency to 9.4%, the KT detachment rate (which is normally 4.8/μm) was reduced to 0.45/μm.
